# Effect of Heptaflourane Inhalation and Anesthesia Induction on Hemodynamics of Elderly Patients Undergoing Elective Gastrointestinal Tumor Surgery

**DOI:** 10.1155/2022/9022614

**Published:** 2022-05-13

**Authors:** Guozhang Ren, Chen Li, Xiaoxia Wei, Jing Wang

**Affiliations:** ^1^Department of Anesthesiology, Yantai Hospital of Traditional Chinese Medicine, Yantai, 264000 Shandong, China; ^2^Department of Anesthesiology, Jinan Second People's Hospital, Jinan, 250001 Shandong, China; ^3^Department of Anesthesiology, Hainan Women and Children's Medical Center, Haikou, 570100 Hainan, China

## Abstract

Gastrointestinal stromal tumors (GISTs) are rare malignancies that begin in specific cells in the GI tract's (also known as the digestive tract's) wall. The microenvironment of gastrointestinal cancers has gotten a lot of interest in the last decade. There are various obstacles connected with providing care to individuals with gastrointestinal cancers, especially the elderly. The physiological reserves of elderly individuals are generally depleted, and comorbidities might limit treatment options and increase problems. Surgeons and anesthesiologists must be aware of the measures that must be used while dealing with this fragile population. Anesthesia is a term that refers to the use of drugs to alleviate pain during the surgery and other treatments. Anesthesia is crucial to a patient's successful treatment and recovery. To induce and maintain general anesthesia in the operating room, inhalation anesthetics (isoflurane, halothane, nitrous oxide, sevoflurane, and desflurane, the most commonly used agents in practice today) are utilized. Inhalation anesthetics are drugs used to give general anesthesia for surgery in the operating room. Anesthetics have the potential to cause substantial cardiac depression as well as hemodynamic instability. In this study, we propose the SBWOA (spark bumper whale optimization algorithm), which is used to assess the patient's risk before surgery. The entire experiment was run through Matlab simulations.

## 1. Introduction

A gastrointestinal stromal tumor (GIST) has gotten a lot of attention in the last ten years due to its unusual biologic behavior, clinicopathological features, molecular causes, and therapeutic implications. It is a major health-care burden throughout the world. The majority of gastrointestinal (GI) cancer research and treatment attempts has focused on cell-autonomous processes in the epithelial compartment. The clinical behavior of gastrointestinal stromal tumors is notoriously variable, making it difficult to determine malignancy and prognosis [1]. Surgery is still the most common treatment for GI stromal tumors; however, the degree of resection, including regional lymph nodes and other organs, is uncertain [2]. Researchers from a range of fields have been studying the relationship between ambient fluoride and human health for over a century. Fluoride dust and gas exposure in the workplace was already related to a higher prevalence of bladder cancer and several respiratory illnesses. Fluoride consumption may also raise the risk of kidney and bladder cancer due to the tendency for hydrogen fluoride, a caustic and possibly poisonous chemical, to develop in urine's acidic environment. In situations of acute fluoride toxicity, GI symptoms such as nausea, vomiting, diarrhea, and abdominal discomfort have been observed [3].

Because of a greater understanding of gut physiology and improved surgical and anesthetic procedures, the practice of gastrointestinal surgery has developed during the previous quarter-century. As a result, perioperative morbidity and death have decreased. Organ dysfunction, infection complications, and delayed convalescence are still related to large elective and nonelective gastrointestinal surgeries [4]. The gastroenterologist used to administer anesthesia for gastrointestinal endoscopic procedures. Complex treatments, on the other hand, are increasingly being performed on very ill patients. As a result, anesthesiologists are increasingly delivering anesthesia and sedation for many of these patients in the gastrointestinal endoscopic suite. Anesthesia can affect the outcome of surgery in a variety of ways, especially for gastrointestinal procedures [5].

Anesthetic drugs and methods, surgical procedures, and the patient's medical comorbidities are all known to cause hemodynamic disturbances. The most fundamental indicators of cardiac health, such as arterial heartbeat and cardiac output, are known as hemodynamics. This study looked at the effect of flourane inhalation and anesthesia induction on the hemodynamics of elderly individuals undergoing elective gastrointestinal stromal tumor surgery. The following is a breakdown of how the research effort will be organized in the future. The literature review and problem statement are presented in Section 2. The techniques and materials of our research work are presented in Section 3. The performance evaluation is included in Section 4, and the paper's conclusion is found in Section 5.

## 2. Related Works

Some of the literary works focus on the effect of fluorine and anesthetic induction on hemodynamic of elderly patients undergoing gastrointestinal tumors. In [6], the author mentions the intraoperative epidural anesthesia effects on QOL (quality of life) and central nervous system damage in aged patients following esophagectomy. The aged patients in this randomized controlled trial were scheduled for thoracoscopic-laparoscopic esophagectomy. The experimental group received general anesthetic as well as epidural anesthesia, while the control group received only general anesthesia. Before being extubated, all patients got the same epidural analgesia treatment at the end of surgery. In [7], the author compared flurbiprofen and nalbuphine against sufentanil and flurbiprofen as part of a multifunctional analgesic regimen for elderly individuals having colorectal treatment for transverse abdominis plane block (TAPB). 158 elderly patients scheduled during regional anesthetic for optional major surgical interventions with TAPB were randomly divided into four groups based on intravenous analgesic concentrations of nalbuphine and flurbiprofen postoperatively (PCIA). At 7, 14, 28, and 36 hours after surgery, pain, effective PCIA number crunching, and side effects were documented. In [8], the author discusses the pharmacology of modern anesthetic medications in elderly patients, neurophysiological changes with aging, and current dosing recommendations for anesthetic drugs. In [9], the author mentioned the effects of several volatile anesthetic medicines on angiogenesis and postoperative metastasis or relapse, as well as their link with immune response and cancer cell biology. In [10], the author investigated the combination of surgery and anesthesia as an important aspect of the treatment of advanced-stage malignancies. The goal of this article is to go over the existing research and emphasize the processes by which local anesthetics are thought to prevent cancer recurrence. In [11], the author mentioned that surgery is the primary treatment for possibly curable solid tumors, metastatic disease that major reason of fatality that occurs due to cancer in individuals who have had previous surgical treatment. In [12], the author demonstrated the connection between surgery and anesthetic-induced immunosuppression and cancer recurrence. It is hypothalamic–pituitary–renal axis and sympathetic nervous system (SNS) stimulation by tumor-derived soluble substances that limit immune system function during surgery and anesthesia. In [13], the author looked into the worldwide experience with GIST detection, prognostic, and therapy, as well as our case series. In [14], the author mentioned that GISTs are a type of gastrointestinal cancer. The stomach is the most commonplace for them. The goal of their aim was to assess the distinctiveness of GISTs in older patients to improve therapy methods and patient survival. In [15], the author wanted to see whether score techniques for hazard analysis may be utilized for operations' audits of aged patients who underwent optional intestinal operation, the author intended to find out. The author's goal in [16] was to explore into the causes of reduced muscle strength in elderly persons with GI tumors, as well as diagnostic indicators and short-term outcomes following gastrointestinal tumor resection, to see whether there is a low lean muscle and relatively brief surgical prognosis are linked. In [17], the author mentions that the incidence of gastrointestinal cancer (GI) rises with each decade of life and is the primary cause of fatality in sufferers over the age of 70. In [18], according to the author's clinical evidence, PIH (postinduction hypotension) is quite common in surgical people treated general anesthesia, and it usually occurs within 20 minutes of the induction of general anesthesia. The author of [9, 19] describes how a variety of circumstances can lead to an increase in cancer-related mortality during the perioperative period. Regrettably, volatile anesthesia may exacerbate the negative consequences. In this paper, we evaluate the impact of several volatile anesthetic drugs on angiogenesis and postoperative metastasis or recurrence, as well as their association with the immune system and cancer cell biology.

### 2.1. Problem Statement

The gastrointestinal stromal tumor can cause one or multiple tumors in those who are affected. GISTs are most frequent in those aged 40 to 70, but they can also happen in children and young adults. Small tumors can be undetected for a long time. Some persons with GISTs, however, may have abdominal swelling and pain, nausea, vomiting, loss of appetite, or weight loss. Tumors can sometimes induce bleeding in the gastrointestinal tract, resulting in low red blood cell counts (anemia) and, as a result, fatigue and exhaustion. Bleeding into the intestines can result in black, tarry feces, whereas bleeding into the throat or stomach might result in blood vomiting. Patients with gastrointestinal stromal tumors are typically treated surgically. Before surgery, many of these patients are now being anesthetized by anesthesiologists. Induction of anesthetics on elderly patients can add additional risk. Postoperative delirium, urosepsis, aspiration, falls, malnutrition, pressure ulcers, adverse drug reactions, and failure to return to ambulation or home are all more common in these patients. In this study, we evaluate the patient's risk condition over surgery using spark bumper whale optimization algorithm (SBWOA).

## 3. Proposed Work

**Figure 1:**
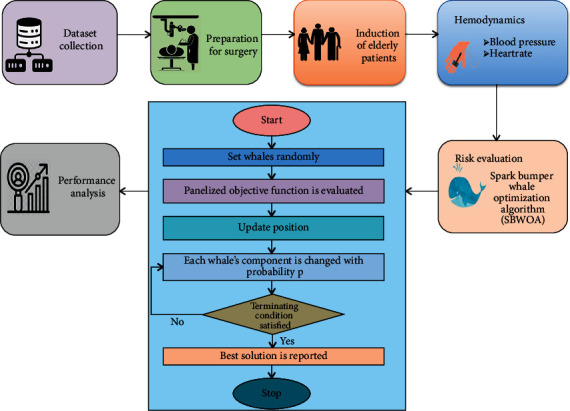
Schematic representation of the suggested methodology.

This study investigates the effects of flourane inhalation and anesthetic induction on the hemodynamics of aged people undergoing elective gastrointestinal tumor surgery. The majority of individuals with gastrointestinal tumors is treated with surgery. The most crucial aspect of the surgical operation is anesthesia induction. Hemodynamic monitoring is used to detect aberrant physiology and act before serious consequences, such as organ failure and death, arise. Hemodynamics determines the cardiovascular, ventilation, and respiration ability of a person's tissue and major organs. The proposed spark bumper whale optimization technique was used to assess the patient's risk state during surgery.  Figure 1 depicts the workflow in detail.

### 3.1. Dataset Collection

This is a randomized controlled study conducted at a single site that was authorized by the Ethics Committee of Yantai Hospital of Traditional Chinese Medicine which was approved on January2019. The research took place at Yantai Hospital of Traditional Chinese Medicine from January to November 2019. This study included patients who were over 65 years old and had a BMI of less than 28 kg/cm^2^ and were scheduled for gastrointestinal tumor excision under general anesthesia. The following were used as exclusion criteria (Table 1):

### 3.2. Preparation for Surgery

The health-care team's ultimate goal is not merely survival. All health-care practitioners have a responsibility to give treatment in a way that protects the patient's physical, emotional, and cognitive integrity during the hospitalization period and after discharge. An interdisciplinary team should evaluate the patient's risk factors, using all available information to make an informed choice about any preoperative interventions, clearly define the scope and type of surgery and anesthesia required, and coordinate postoperative care, such as rehabilitation, analgesia, and nutritional support. Substance misuse, diabetes, and inflammatory bowel disease are all risk factors for gastrointestinal malignancies that must be detected (and treated) before surgery. It has been proven that abstaining from alcohol and nicotine for at least one month before surgery reduces the risk of complications [20].

Many intraoperative and postoperative parameters can be altered favorably by early perioperative decisions and treatments, greatly increasing the chances of effective treatment of older cancer patients. Premedication, fasting, hydration management, and antibiotics are just a few of the aspects to consider. Before induction, fasting should be limited to 6 hours without solid meals and two hours without clear fluids. Furthermore, preoperative fluid restrictions were the strongest predictor of postoperative delirium in the recovery room (POD). Malnutrition and dehydration are common in senior citizens due to a weakened sense of taste and thirst, and gastrointestinal cancers can exacerbate the situation by causing nausea, chemotherapy side effects, or a longer stomach clearing time. Fasting times might be substantially longer, especially for operations scheduled early in the morning. Prolonged fasting can aggravate diabetes and cardiovascular diseases; thus, it is critical to encourage the patient to stick to the authorized fasting period and to avoid or notify any schedule delays as soon as possible.

Intravenous fluids should be considered for at-risk elderly individuals as well as patients who are having significant surgery delays. Preoperative carbohydrate loading the night before and 2–3 hours before the procedure has been demonstrated to improve outcomes in small studies. Premedication with benzodiazepines should be avoided if at all possible, as it was already related to a higher chance of postoperative problems like delirium. Individuals with extreme anxiety should not be denied premedication, as untreated patients are more likely to develop problems.

Dosage changes should be explored in such circumstances. Be aware of potential side effects, such as respiratory suppression, and keep premedicated patients under constant observation. Premedication, as well as age, can alter the reference values; therefore, electroencephalography (EEG) monitoring should commence before induction of anesthesia in all patients. Antibiotics should be given between 30 minutes and 1 hour before surgery, either on the ward or immediately upon arriving in the operating room, to lower the risk of postoperative infections. Furthermore, due to diminished muscle mass or impaired circulation, aged individuals' thermoregulation can be disturbed, and they have a restricted compensatory mechanism. Because even mild hypothermia has been known to disrupt clotting and raise postoperative infection rates, they should be covered and forcefully warmed as soon as they arrive, even before a peridural catheter is put.

### 3.3. Induction of Elderly Patients

Anesthesia induction for older patients must take into account their existing physical condition and comorbidities. While regional anesthesia is not an option for gastrointestinal surgery, a patient-controlled epidural analgesia device can help cut down on the number of opioids needed during and after the procedure. The systems also allow for faster movement, more independence from personnel during the surgical phase, and intestinal stimulation via sympathetic innervation inhibition. Lower muscle and fat proportions, as well as reduced neuronal mass, dehydration, and organ dysfunctions, can significantly affect medication distribution and metabolism. Because of the shift in drug pharmacodynamics and pharmacokinetics, normal dosages in senior oncologic patients can be hazardous. This altered state must be expected, and the chemical selection and dosage should be adjusted to avoid difficulties. Induction and maintenance of anesthesia should be done if possible.

Even though high inspired doses (~5%) were supplied from the start, sevoflurane induction was quick and without respiratory discomfort. The vaporizer's anesthetic output may have reduced the speed of induction (109 ± 25 s) (maximum setting of 5 percent). To reduce the hemodynamic reaction to tracheal intubation, all patients were given a minor dose of fentanyl before anesthetic induction. If an opioid analgesic had not been given before induction of anesthesia, inhaled sevoflurane induction would probably have been less well tolerated. Respiratory depression tended to develop with increasing end-tidal sevoflurane concentration during inhaled sevoflurane induction. The cardiovascular depression caused by sevoflurane induction was much smaller than that caused by propofol (2 mg/kg IV) induction.

### 3.4. Hemodynamics

Anesthetic drugs and methods, surgical manipulations, and the patient's medical comorbidities are all known to cause hemodynamic disturbances. Hemodynamics is the study of the allocation of pressures and fluxes in the circulatory system. The term “pressure” refers to hydrostatic pressure, which is an isotropic compressive stress with force units per unit area. The pumping heart supplies internal energy to the blood by pressurizing it, allowing it to move through the circulation. The idea of a blood vessel's viscous flow resistance, which is described as the ratio of pressure drop *p* to volume flow rate *V*, is shown in

(1) $$\begin{array}{c} R=\frac{∆p}{V}. \end{array}$$

The flow rates in each component of the network can be calculated using fundamental principles such as the rules for the combined resistance of resistors connected in series or parallel.

(2) $$\begin{array}{c} R_{s}=R_{1}+R_{2}\text{ and }R_{p}=\frac{R_{1R_{2}}}{R_{1}+R_{2}}, \end{array}$$

where *R*_*s*_ and *R*_*p*_ are the series and parallel combinations of effective resistance, and *R*_1_ and *R*_2_ are the two resistances. The flow resistance concept can be adapted to the entire peripheral circulation as a single resistance, yielding

(3) $$\begin{array}{c} \text{TP}=\frac{\text{MP}-\text{CP}}{\text{CO}}, \end{array}$$

where total peripheral resistance is TP, mean arterial pressure is MP, central venous pressure is CP, and cardiac output is CO. 2/3 of diastolic BP+ 1/3 of systolic BP is a common definition for the MP, which approximates the temporal mean of arterial pressure. TP is determined by the geometric parameters of the vascular system, including the vascular tone effect on artery diameter, as well as the blood flow properties, at any given time. It specifies how much pressure the left heart must produce to provide a certain level of cardiac output. The resistance of the pulmonary circulation can be calculated using a similar formula:

(4) $$\begin{array}{c} \text{PVR}=\frac{\text{MPA}-\text{PW}}{\text{CO}}, \end{array}$$

where PVR is for pulmonary vascular resistance, MPA stands for mean pulmonary arterial pressure, which is measured similarly to MP, and PW stands for pulmonary wedge pressure. The PW is calculated by inserting insertion of a respiratory catheterization with an inner tube into a minor segment of the pulmonary vein and measurement of flow downstream of the blockage. It calculates the pulmonary venous pressure. The pressure decrease across the lungs (normally around 10 mmHg) is far less than the pressure drop across the systemic circulation (typically about 100 mmHg). PVR is approximately one-eighth the size of systemic TP, though there can be large variances.

The circulatory system's primary function is to ensure that blood flow is distributed evenly throughout the body to meet the tissues' fluctuating needs for oxygen and other nutrients, as well as to remove waste products.

#### 3.4.1. General Considerations for Blood Pressure Management

During anesthesia, standard blood pressure (BP) measures are taken at least every five minutes using an automated noninvasive oscillometric BP cuff. An intra-arterial catheter is utilized in a few patients, especially those who need continuous monitoring. Other noninvasive methods, such as noninvasive continuous finger cuff readings, are employed less frequently.

BP should be maintained within 20% of the patient's baseline and keep mean arterial pressure (MAP) ≥ 65 mmHg (and systolic BP ≥ 100 mmHg) to avoid myocardial injury following noncardiac surgery (MINS) or myocardial infarction (MI), central nervous system (CNS), acute kidney injury (AKI), ischemic events, and mortality or ischemic events. The normal boundary of vasodilatation of blood flow to the brain in normotensive adults is a MAP of 70 mmHg or above, albeit this lower limit and the blood flow reserve that can temporarily buffer the CNS against hypotension vary greatly by individual. To avoid serious cardiovascular events or organ failure, many individuals may require an intraoperative MAP target greater than 65 mmHg.

#### 3.4.2. General Considerations for Heart Rate Management

In general, we try to avoid tachycardia by keeping our HR (heart rate) below 100 beats per minute. We keep a lower HR in patients with ischemic heart disease (e.g., 50 to 80 bpm) because tachycardia impairs both myocardial oxygen supply and demand. Severe bradycardia is addressed if the heart rate is less than 40 beats per minute, is coupled with transitory asystole, or is hemodynamically significant with indicators of poor perfusion (e.g., hypotension and electrocardiographic evidence of ischemia).

MINS (OR 1.27, 95 percent CI 1.07-1.50) and MI (OR 1.34, 95 percent CI 1.05-1.70), as well as death, were linked to intraoperative tachycardia with an HR >100 bpm (OR 2.65, 95 percent CI 2.06-3.41). If the tachycardia lasted longer than 30 minutes, there was a greater chance of MINS (OR 2.22, 95 percent CI 1.71-2.88). Slow intraoperative HR 55 bpm, on the other hand, was linked to a lower risk of MINS (OR 0.70, 95 percent CI 0.59-0.82), MI (OR 0.75, 95 percent CI 0.58-0.97), and mortality (OR 0.58, 95 percent CI 0.41-0.81), with a trend toward a lower risk of MINS with increasing duration of slow recorded HR 55 bpm.

Prevention and treatment of tachycardia and bradycardia depend on the likely cause, the time of day, and the patient's baseline condition. The same is true for intraoperative blood pressure.

*(1) Atrial Tacharrrhythmias*. The most common type of intraoperative tachycardia is sinus tachycardia, which has an HR of more than 100 beats per minute (bpm).

*(2) Atrial Bradyarrhythmias*. Sinus bradycardia is defined as intraoperative bradycardia with an HR of fewer than 60 beats per minute (bpm).

### 3.5. Risk Evaluation

Over the last century, advances in modern health care have considerably increased the average lifespan around the world, and the elderly represent the fastest-growing population in healthcare. Each year, an increasing number of people undergo anesthesia for surgery and other procedures. We suggested the spark bumper whale optimization algorithm in this section for a patient's risk situation during surgery.  Table 2 shows cases where an operation was canceled following anesthesia and the reasons for cancellation.

The humpback whale's bubble net foraging activity inspired the spark bumper whale optimization algorithm (SBWOA). The humpback whale first surrounds the victim with a vast number of spiral bubbles before hunting it. Humpback whales adopt 2 methods for poaching: the first is to reduce the encircling technique; another is to use the spirals updated location approach. These two methods are used in tandem in each predation. The program creates the possibility of a random assortment  p(p ∈ [0, 1]) to emulate these two options. The humpback whale preys on the spiral position approach *p* ≥ 0.5; when *p* < 0.5, the vulnerability of the surrounding system is dwindling technique. The whales randomly look for the high danger according to their location in the shrinking encircling mechanism's approach. The algorithm introduces the coefficient vector $\overset{⟶}{B}$ to illustrate the algorithm's randomness. When | $\overset{⟶}{B}$ | > 1|, a random search agent is picked, while when |$\overset{⟶}{B}$| < 1, the highest risk solution is chosen for updating the position of the search agents. It is easy to identify the global optimal solution since it has numerous search strategies, which is a significant benefit over the standard optimization algorithm. The following is the algorithm's key mathematical model:

(5) $$\begin{array}{c} \overset{⟶}{F}=\left|\overset{⟶}{B}.{\overset{⟶}{Y}}^{*}\left(t\right)-\overset{⟶}{Y}\left(t\right)\right|, \end{array}$$

where *t* is the iteration current number |$\overset{⟶}{Y}$|; *Y*∗ denotes the vector's position and the best solution position vector found thus far, respectively; $\overset{⟶}{B}$ denotes the vector coefficiency; and $\overset{⟶}{F}$denotes the search distance. A random search agent's definition

(6) $$\begin{array}{c} Y\left(t+1\right)=Y_{\mathrm{rand}}-\overset{⟶}{B}.\overset{⟶}{F} \end{array}$$

where $\overset{⟶}{B}$ is the coefficient vector and *Y*_rand_ is the random coordinates (a random whale) chosen from the resident generation. When a humpback whale hunts, it uses the spiral updating position technique, which has the following spiral equation:

(7) $$\begin{array}{c} \overset{⟶}{Y}\left(t+1\right)=\overset{⟶}{\overset{´}{F}}.e^{ml}.\cos\left(2\pi l\right)+{\overset{⟶}{Y}}^{*}\left(t\right), \end{array}$$

where _*F*_^⟶^′ is the *i*^*th*^ whale distance fromthe prey (best answer so far); the logarithmic spiral's shape is defined by the constant *m*; random number *l* is in the range [1]; and ∗denotes the multiplication of elements by elements.

Adverse reactions to FLURANE include respiratory depression, hypotension, and arrhythmias. Adverse reactions to FLURANE are often dosage-dependent expansions of pharmacophysiologic effects. In the postoperative period, shivering, nausea, vomiting, and ileus have all been reported. It is difficult to prevent a change in the patient's physical status during general anesthetic induction, such as rapid drug-related anaphylactic shock, arrhythmias, and hypoxemia induced by atelectasis. The spark bumper whale optimization algorithm is used to assess these hazards.

#### 3.5.1. Anaphylactic Shock

Anaphylaxis is the word for a life-threatening allergic reaction. It causes a rash, a rapid heart rate, and anaphylactic shock, which is characterized by a rash, low pulse rate, and shock. Patients get an anaphylactic shock as a result of anesthetic induction. When surgeons learn that a patient is suffering from anaphylactic shock, they cancel the surgery.

#### 3.5.2. Arrhythmias

When a patient is given anesthesia, one of the most common cardiovascular complications is arrhythmia. According to reports, 70% of people who receive general anesthesia for various surgical operations feel it. Arrhythmia is more common in patients undergoing cardiac surgery.

#### 3.5.3. Hypoxemia

Hypoxemia is one of the most serious dangers that patients experience during anesthesia and surgery. Reduced oxygen levels in the circulation, especially in the artery, are called hypoxia. It is a sign of a respiratory or circulatory issue that can cause a variety of symptoms, including suffocation in breathing.

## 4. Result and Discussion

In this section, a graphical representation of the effects of anesthesia induction on a patient is presented. The survival rate is the percentage of people in a research or therapy team who are still surviving after a particular period of time after diagnosis.

**Figure 2:**
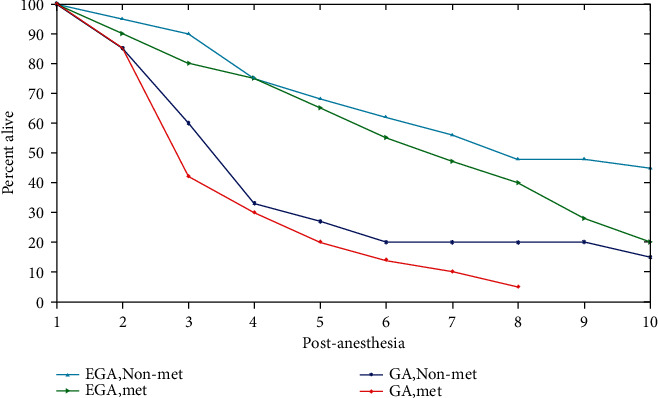
Survival rate.

The survival rate of a patient who has had anesthesia is shown in Figure 2. Kaplan-Meier survival rates are stratified by pathogenesis stage and kind of anesthesia. Patients without metastases had a longer median survival (6.1 years). Epidural-supplemented anaesthesia (EGA); nonmetastasis (nonmet); metastasis (MET), unsupplemented general anesthesia (GA) are compared to other patients, those who have had epidural-supplemented anesthesia without metastasis have the highest survival rate. When compared to other patients, those who have had epidural-supplemented anesthesia without metastasis have the highest survival rate.

**Figure 3:**
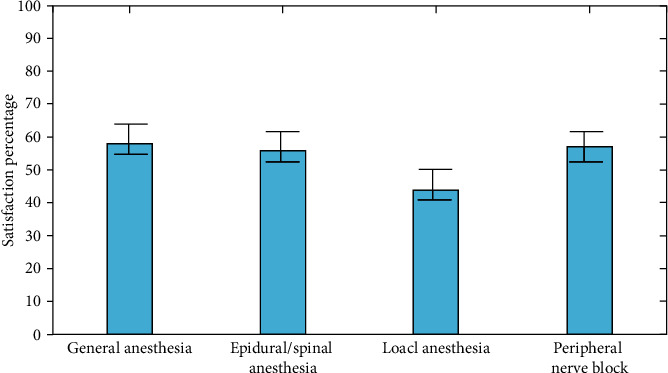
Satisfaction percentages grouped by type of anesthesia.

The average percentage of people who are satisfied varies depending on the type of anesthetic. With 57 percent, general anesthesia had the highest average overall satisfaction. Local anesthetic instances, on the other hand, had the lowest percentages, with an average of only 41%.  Figure 3 shows the satisfaction percentages broken down by anesthetic type.

**Figure 4:**
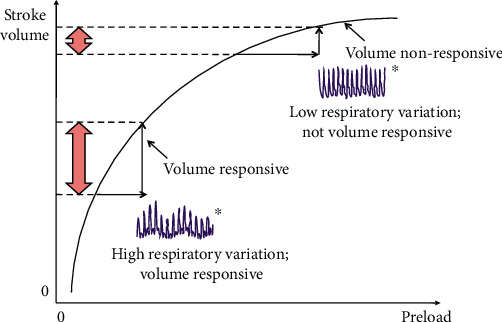
Hemodynamic management during anesthesia.

Anesthetic drugs can produce considerable cardiac depression and hemodynamic instability even in healthy patients undergoing simple surgeries. Almost all anesthetics have inherent cardiac depressive effects, but some may be disguised by sympathetic activation. Hemodynamic instability occurs when blood flows too quickly through the heart, causing the cardiac function to become unstable. As a result, hemodynamic control is critical for individuals who have had anesthetic induction. Hemodynamic control during anesthesia is depicted in Figure 4.

**Figure 5:**
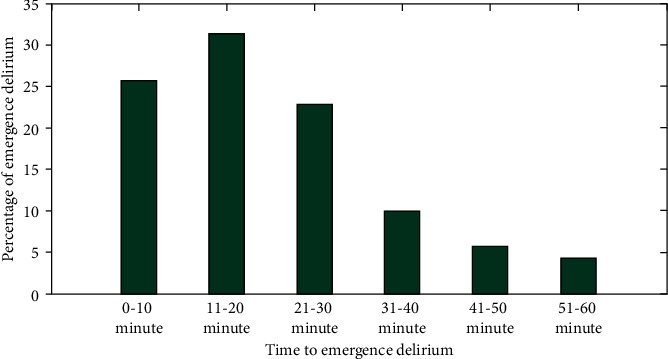
Emergence of delirium after the induction of anesthesia.

Figure 5 shows a bar graph showing the proportion and time it takes for elderly individuals to develop delirium following anesthesia in a post-anesthesia care unit with 31.4 percent of patients experiencing emerging delirium within the first 11-20 minutes.

## 5. Conclusion

In most investigations, GIST was found in 10-15 million per year. Interestingly, China's Shanxi province has the lowest incidence rate of 4.3 per million per year. In China (Hong Kong and Shanghai regions), Taiwan, and Norway (Northern part), incidence rates of 19-22 million per year were observed. During surgery and other procedures, anesthesia is used to alleviate pain. Even if the patient has substantial health issues, general anesthesia will most likely be tolerated without incident. When compared with different types of anesthesia, general anesthesia with 57% had the highest average overall satisfaction. They may, however, experience some danger with any medicine or medical procedure. The spark bumper whale optimization algorithm (SBWOA) is used to assess the risk of a patient's condition over surgery. The use of sevoflurane for anesthetic induction is quick and has few negative effects. When compared to isoflurane, sevoflurane resulted in speedier recovery from anesthesia. Finally, we urge that further research be conducted with large sample size and a longitudinal follow-up study.

## Figures and Tables

**Algorithm 1:**
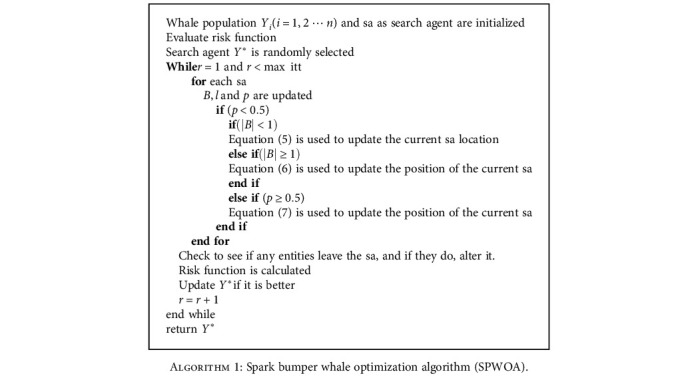
Spark bumper whale optimization algorithm (SPWOA).

**Table 1:** Characteristics of patient.

Variables	Groups	*P* value
Age (*x*)	72.30 ± 5.26	0.411
Sex		
Male	70 (82.5%)	0.071
Female	50 (62.5%)	
Height in cm	164.66 ± 7.45	0.078
Weight in kg	63.83 ± 9.84	0.796
BMI in kg/*cm*^2^	23.50 ± 2.79	0.510
Operation type		
Stomach	31 (39.2%)	0.236
Colon	21 (26.6%)	
Rectum	27 (34.2%)	
History of smoking	30 (37.5%)	0.606
History of drinking	26 (32.5%)	0.299
Diabetes	7 (8.8%)	0.169
Hypertension	19 (23.8%)	0.258
Baseline blood pressure	124.66 ± 12.99	0.576
SBP (mmHg)	74.29 ± 8.67	0.386
DBP (mmHg)	90.59 ± 9.23	0.368

**Table 2:** Surgery canceled cases after anesthesia.

Reason for surgery cancellation	Total (*n* = 12)
Anaphylactic shock	3
Arrhythmias	
Tdp	1
CAVB	1
Arterial fibrillation	1
Cardiac arrest	1
Anemia	1
Hypoxemia	1
Other	3

## Data Availability

The datasets used and/or analyzed during the present study can be available from the corresponding author if needed.
